# Differentiation of Acute Q Fever from Other Infections in Patients Presenting to Hospitals, the Netherlands^1^

**DOI:** 10.3201/eid2108.140196

**Published:** 2015-08

**Authors:** Stephan P. Keijmel, Elmer Krijger, Corine E. Delsing, Tom Sprong, Marrigje H. Nabuurs-Franssen, Chantal P. Bleeker-Rovers

**Affiliations:** Radboud university medical center, Nijmegen, the Netherlands (S.P. Keijmel, E. Krijger, C.P. Bleeker-Rovers);; Medical Spectrum Twente, Enschede, the Netherlands (C.E. Delsing);; Canisius Wilhelmina Hospital, Nijmegen (T. Sprong, M.H. Nabuurs-Franssen)

**Keywords:** Q fever, acute Q fever, chronic Q fever, Coxiella burnetii, bacteria, case–control study, clinical practice, hospital, prophylaxis, prophylactic treatment, the Netherlands

## Abstract

Differentiating acute Q fever from infections caused by other pathogens is essential. We conducted a retrospective case–control study to evaluate differences in clinical signs, symptoms, and outcomes for 82 patients with acute Q fever and 52 control patients who had pneumonia, fever and lower respiratory tract symptoms, or fever and hepatitis, but had negative serologic results for Q fever. Patients with acute Q fever were younger and had higher C-reactive protein levels but lower leukocyte counts. However, a large overlap was found. In patients with an indication for prophylaxis, chronic Q fever did not develop after patients received prophylaxis but did develop in 50% of patients who did not receive prophylaxis. Differentiating acute Q fever from other respiratory infections, fever, or hepatitis is not possible without serologic testing or PCR. If risk factors for chronic Q fever are present, prophylactic treatment is advised.

## Abstract 2

Distinguishing acute Q fever from respiratory infections, fever, or hepatitis requires serologic testing or PCR.

Q fever is a zoonosis caused by the bacterium *Coxiella burnetii*. During 2007–2010, the southern part of the Netherlands had the largest outbreak of Q fever ever reported (*1**,**2*). Infection with *C. burnetii* is symptomatic in ≈40% of all patients (*3*). Clinical signs range from a mild influenza-like illness to pneumonia or a hepatitis-like syndrome and can differ by region (*4**,**5*). After initial infection, chronic Q fever will develop in 1%–5% of patients (*1**,**3*). Furthermore, long-lasting fatigue will develop in ≈20% of all patients with symptomatic acute Q fever (*6**–**8*) without development of chronic Q fever (*9*).

Treatment for acute infection decreases the duration of fever, increases recovery from pneumonia (*10*), and might lead to a lower percentage of patients in whom chronic Q fever will develop (*10**–**13*). In addition, several reports indicate that, in acute Q fever patients at risk for development of chronic Q fever, prophylactic treatment might prevent persistent infection (*12**,**14*). Therefore, recognizing Q fever in an early stage is a useful strategy.

The only available data on symptoms of acute Q fever in the Netherlands were obtained from a retrospective study that collected data several months after onset of disease by sending questionnaires to patients with acute Q fever (*15*). However, this method for obtaining data is limited by a high risk for recall bias. To help physicians differentiate acute Q fever from other diseases, a clear description of signs and symptoms compatible with *C. burnetii* infection is desirable.

The purpose of this case–control study was to evaluate differences in clinical signs and symptoms between patients with acute Q fever referred to a hospital and a control group of patients with signs and symptoms that led to addition of Q fever in the differential diagnosis. Furthermore, outcome of patients hospitalized with acute Q fever were evaluated, and the effect of prophylactic treatment for those patients with an indication to prevent development of chronic Q fever was analyzed.

## Materials and Methods

### Patients

The study group consisted of adult patients who came to the Radboud university medical center or Canisius Wilhelmina Hospital in Nijmegen, the Netherlands, during January 2007–March 2011 with pneumonia, fever and lower respiratory tract symptoms, or fever and hepatitis, and who were given a diagnosis of acute Q fever. Symptoms had to be present for <3 weeks before presentation. Exclusion criteria were chronic Q fever and a known previous acute Q fever episode. The same clinical criteria were used for the control group, but Q fever serologic results and, if available, PCR results had to remain negative. A standardized case report form was completed for every patient. According to national law, this study was exempt from approval by an ethics committee because of the retrospective characteristics of the study and the anonymous storage of data.

### PCR and Serologic Analysis

During January 2007–March 2011, several laboratory techniques were used to diagnose acute Q fever. Because both hospitals collaborate extensively, the same microbiological laboratory techniques were used in both hospitals. The PCR used to detect DNA of *C. burnetii* in serum was an in-house, real-time PCR directed against insertion sequence IS1111a.

Serologic analysis was performed for blood samples by using the *Coxiella burnetii* (Q Fever) IgM ELISA (PanBio Pty Ltd., Windsor, Queensland, Australia), which detects IgM against phase II antigens and has a cutoff index of 1.1; a complement fixation assay (CFA) (Virion-Serion, Würzburg, Germany), which detects *C. burnetii* phase II antigens and shows a positive result if the titer is >1:10; and a Q fever immunofluorescent assay (IFA) for IgG and IgM (Focus Diagnostics Inc., Cypress, CA, USA), which detects IgM and IgG against phase I and phase II antigens and shows a positive result if the titer is >1:16.

### Definition of Acute Q Fever

On the basis of the algorithm published by the Dutch working group on diagnostics of acute Q fever (*16*), the following definition of acute Q fever was used for all included patients: pneumonia, lower respiratory tract symptoms and fever, or hepatitis-like symptoms and fever, all <3 weeks before presentation; and 1) a positive serum PCR result <21 days of onset of disease; or 2) a negative serum PCR result, but a positive ELISA result for IgM against phase II antigens of *C. burnetii* and a positive CFA result for immunoglobulins against *C. burnetii*; or 3) a negative serum PCR result but a positive ELISA result and a positive IFA result for IgM and IgG against phase I and phase II antigens of *C. burnetii*; or 4) two serum samples tested by CFA or IFA during an interval of >2 weeks that showed seroconversion or a 4-fold increase in titer. 

A blood sample for Q fever serologic analysis obtained >2 weeks after the first day of illness was required because it was not possible to rule out acute Q fever if serologic samples are taken only at an earlier point, even if PCR results were negative during that period (*16*). Patients were selected only if an appropriate diagnostic procedure for Q fever was performed.

### Treatment

Adequate treatment for acute Q fever was defined as antimicrobial drug therapy with doxycycline (200 mg/d), moxifloxacin (400 mg 1×/d), or ciprofloxacin (500 mg 2×/d) for >14 days (*17**,**18*). Indications for prophylactic treatment to prevent development of chronic Q fever were patients who met the criteria for endocarditis prophylaxis according to the international guidelines of the American Heart Association (*19*); patients with a structural aortic valve defect or mitral valve defect (*12*); patients with a known aneurysm of the aorta or other large vessels; and patients with a vascular prosthesis. Adequate prophylactic treatment was defined as doxycycline (200 mg/d) and hydroxychloroquine (200 mg 3×/d) for >6 months.

### Statistical Methods

All data were analyzed by using SPSS version 20.0 (IBM, Armonk, NY, USA). For analysis of qualitative data, the Pearson’s χ^2^ test was used. To evaluate the effect of prophylactic treatment, the Barnard exact test was used because this test is more powerful than the Fisher exact test for instances of smaller sample sizes (*20*). For quantitative data, the Student *t*-test was used. A p value <0.05 was considered significant.

## Results

### General Characteristics

A total of 82 patients with acute Q fever who fulfilled inclusion criteria for the study group and 52 patients who fulfilled criteria for the control group were included in the study (Table 1). Patients with acute Q fever were younger (mean ± SD age 52 ± 16 years vs. 59 ± 16 years; p = 0.03); had less often a history of lung disease (p = 0.001); and were immunocompromised less often (p = 0.002). Patients with acute Q fever had more history of smoking (p = 0.01) and a higher frequency of a sore throat (p = 0.008) (Table 2). Production of sputum was reported less frequently by patients with acute Q fever (p = 0.049).

**Table 1:** Characteristics for patients with acute Q fever and control group with negative serologic results for Q fever, the Netherlands*

Characteristic	Study group	Control group	p value
No. patients	82	52	NS†
Male sex, no. (%)	53 (65)	38 (73)	NS‡
Mean ± SD age, y (range)	52 ± 16 (23–91)	59 ± 16 (19–85)	0.027†
Mean no. days between first day of sickness and presentation	5.5	5.4	NS†
History of lung disease	8/78 (10)	18/51 (35)	0.001‡
Immunocompromised§	5/81 (6)	13/51 (25)	0.002‡
Valvular dysfunction	8/82 (10)	3/52 (6)	NS‡
Valve prosthesis	3/82 (4)	0/52 (0)	NS‡
Aneurysm	2/82 (2)	3/52 (6)	NS‡
Vascular prosthesis	3/82 (4)	3/52 (6)	NS‡
Liver disease	1/82 (1)	1/52 (2)	NS‡
Malignancy	2/82 (2)	9/52 (17)	0.002‡
Diabetes	9/82 (11)	7/52 (13)	NS‡
Contact with cattle	29/47 (62)	8/20 (40)	NS‡
History of smoking	58/74 (78)	25/44 (57)	0.013‡
Alcohol use	17/44 (39)	12/27 (44)	NS‡
Illicit drugs	4/35 (11)	0/18 (0)	NS‡
Proton pump inhibitors¶	13/82 (16)	22/52 (42)	0.001‡
Corticosteroids¶	5/82 (6)	10/51 (20)	0.017‡

*Values are no. positive/no. tested (%) unless otherwise indicated. NS, not significant. †By Student *t-*test. ‡By χ^2^ test. §Also includes patients using corticosteroids. ¶Only medications that differed significantly between groups is shown.

**Table 2:** Signs and symptoms for patients with acute Q fever and control group with negative serologic results for Q fever, the Netherlands*

Characteristic	Study group, n = 82, no. positive/no. tested (%)	Control group, n = 52, no. positive/no. tested (%)	p value†
Fever	64/75 (85)	37/49 (76)	NS
Chills	31/42 (74)	16/28 (57)	NS
Myalgia	22/24 (92)	11/14 (79)	NS
Night sweats	12/19 (63)	9/17 (53)	NS
Weight loss	11/26 (42)	7/14 (50)	NS
Chest pain	11/55 (20)	13/38 (34)	NS
Dyspnea	37/65 (57)	31/43 (72)	NS
Rhinorrhea	1/12 (8)	7/14 (50)	NS
Sore throat	12/22 (55)	1/12 (8)	0.008
Cough	49/76 (64)	38/48 (79)	NS
Sputum production	18/73 (25)	20/48 (42)	0.049
Nausea	14/48 (29)	12/37 (32)	NS
Vomiting	17/47 (36)	10/39 (26)	NS
Abdominal pain	9/51 (18)	6/33 (18)	NS
Diarrhea	9/50 (18)	4/36 (11)	NS
Headache	38/54 (70)	21/27 (78)	NS
Weakness	9/21 (43)	1/9 (11)	NS
Painful joints	7/20 (35)	2/16 (13)	NS
Arthritis	0/17 (0)	1/16 (6)	NS

*NS, not significant. †By χ^2^ test.

### Physical Examination

Of patients with acute Q fever, 18% had shortness of breath (Table 3) compared with 44% in the control group (p = 0.03). A total of 4% of patients with acute Q fever had rhonchi at pulmonary examination compared with 22% in the control group (p = 0.005). Oxygen saturation was significantly higher in patients with acute Q fever (p = 0.02).

**Table 3:** Physical examination results for patients with acute Q fever and control group with negative serologic results for Q fever, the Netherlands*

Characteristic	Study group, n = 82	Control group, n = 52	p value
Dyspnea	13/73 (18)	18/41 (44)	0.03†
Abnormal heart sounds	1/80 (1)	0/51 (0)	NS†
Cardiac murmur	11/80 (14)	4/50 (8)	NS†
Decreased breath sounds	6/78 (8)	7/46 (15)	NS†
Bronchial breath sounds	9/64 (14)	5/37 (14)	NS†
Crackles	36/76 (47)	19/43 (44)	NS†
Rhonchi	3/68 (4)	9/41 (22)	0.005†
Palpable liver	1/69 (1)	1/39 (3)	NS†
Palpable spleen	0/68 (0)	0/36 (0)	NS†
Exanthema	2/9 (22)	0/6 (0)	NS†
Lymphadenopathy	2/27 (7)	2/21 (10)	NS†
Temperature, °C (no. patients)	38.4 (67)	38.3 (48)	NS‡
Heart rate, beats/min (no, patients)	93 (73)	91 (50)	NS‡
Systolic blood pressure, mm Hg, (no. patients)	134 (73)	138 (49)	NS‡
Respiratory rate, breaths/ min (no. patients)	25 (24)	25 (21)	NS‡
Saturation, % oxygenation (no. patients)§	97 (57)	95 (34)	0.022‡

*Values are no positive/no. tested (%) unless otherwise indicated. NS, not significant. †By χ^2^ test. ‡By Student *t-*test. §Saturation without oxygen.

### Laboratory Values

Patients with acute Q fever had a higher levels of C-reactive protein (mean 167 mg/L vs. 117 mg/L; p = 0.02) (Table 4) and lower leukocyte counts (mean 9.0 × 10^9^ cells/L vs. 11.5 × 10^9^ cells/L; p = 0.006). Leukocyte counts remained significantly lower in the first 3 days after presentation (p = 0.006–0.043). At admission to the hospital, no differences were found between the groups for levels of alkaline phosphatase and γ-glutamyl transpeptidase. However, from day 1 onward, levels of alkaline phosphatase and γ-glutamyl transpeptidase were significantly higher in patients with acute Q fever (p = 0.01–0.047 and p = 0.007–0.05, respectively).

**Table 4:** Laboratory values for patients with acute Q fever and control group with negative serologic results for Q fever, the Netherlands*

Laboratory value	Day†	Study group, n = 82		Control group, n = 52		p value‡
		Mean	No. tested	Mean	No. tested	
Hemoglobin, mmol/L; reference range: men 8.1–10.7 mmol/L, women 7.3–9.7 mmol/L	0	8.3	77	8.0	51	NS
	1	7.4	28	7.3	34	NS
	2–3	7.7	27	7.0	29	0.036
	4–6	7.6	27	7.0	29	NS
Leukocytes, × 10^9^ cells/L; reference range 3.5–11.0 × 10^9^ cells/L	0	9.0	80	11.5	50	0.006
	1	8.5	40	10.8	28	0.043
	2–3	8.0	34	11.1	33	0.021
	4–6	10.9	28	9.2	31	NS
Platelets, × 10^9^/L; reference range 20–350 × 10^9^/L	0	239	78	208	50	NS
	1	242	23	178	29	0.038
	2–3	229	19	172	26	0.042
	4–6	298	24	208	27	0.011
Total bilirubin, μmol/L; reference value <17 μmol/L	0	14	26	16	20	NS
	1	12	14	14	8	NS
	2–3	9	12	28	6	0.017
	4–6	8	12	9	6	NS
AP, U/L; reference value <120 U/L	0	104	75	85	50	NS
	1	127	19	75	12	0.047
	2–3	126	26	66	12	0.010
	4–6	145	23	95	15	0.036
ALT, U/L; reference value <45 U/L	0	45	76	37	49	NS
	1	64	22	58	16	NS
	2–3	66	30	40	13	0.050
	4–6	81	22	84	18	NS
γ-GT, U/L; reference value: men <50 U/L, women <35 U/L	0	74	68	65	49	NS
	1	117	21	53	12	0.030
	2–3	106	27	42	9	0.007
	4–6	112	22	66	14	0.050
CRP, mg/L; reference value <10 mg/L	0	167	79	117	50	0.015
	1	184	44	150	37	NS
	2–3	132	46	147	32	NS
	4–6	76	41	98	27	NS
Urea, mmol/L; reference value 2.5–7 mmol/L	0	6.4	79	8.6	51	0.039
	1	6.4	33	7.9	35	NS
	2–3	5.4	38	8.7	35	0.014
	4–6	5.8	34	9.3	30	0.018
Creatinine, μmol/L; reference value: men <110 μmol/L, women <90 μmol/L	0	86	80	105	52	0.042
	1	84	38	103	38	NS
	2–3	79	37	103	37	NS
	4–6	81	36	136	31	NS

*NS, not significant; AP, alkaline phosphatase; ALT, alanine aminotransferase; γ-GT, γ-glutamyl transpeptidase; CRP, C-reactive protein. †Day 0 is the day of coming to the hospital. ‡By Student *t-*test.

### PCR and Serologic Analysis

Serum PCR for DNA of *C. burnetii* was performed for 41 patients in the study group (Table 5). Blood samples were obtained at day 8 ± 7 (mean ± SD) of illness. The sensitivity of this PCR was 56%. For 4 patients, a second blood sample was obtained at day 12 ± 5 of illness. The sensitivity of this PCR was 25%.

**Table 5:** PCR and serologic results for patients in study group with acute Q fever and control group with negative serologic results for Q fever, the Netherlands*

Characteristic	Study group, n = 82	Control group, n = 52	Day of illness for study group, mean ± SD	Day of illness for control group, mean ± SD	Sensitivity, %
PCR					
First sample	23/41	0/15	8 ± 7	8 ± 7	56
Second sample	1/4	0/1	12 ± 5	30 ± 0	25
ELISA					
First sample	20/33	0/18	10 ± 8	7 ± 6	61
Second sample	15/18	0/2	20 ± 11	25 ± 8	83
CFA					
First sample	18/81	0/52	9 ± 19	8 ± 6	22
Second sample	27/34	0/28	18 ± 9	20 ± 12	79
Third sample	5/5	0/3	21 ± 6	26 ± 5	100
Culture					
Blood†	0/42 (0)	0/40 (0)	NA	NA	NA
Urine†	0/30 (0)	0/37 (0)	NA	NA	NA
Sputum‡	1/15 (7)	3/22 (14)	NA	NA	NA
Chest radiograph§	62/79 (78)	28/52 (54)	NA	NA	¶

*Values are no. positive/no. tested (%) unless otherwise indicated. CFA, complement fixation assay; NA, not applicable. †Includes only results for first cultures obtained after coming to the hospital. ‡Includes only results for first cultures obtained after coming to the hospital. In the study group, 1 patient was positive for parainfluenza virus. In the control group, 1 patient was positive for *Moraxella catarrhalis,* 1 patient was positive for *Legionella pneumophila*, and 1 patient was positive for *Streptococcus pneumonia* and *Staphylococcus aureus*. §Includes only first chest radiographs after coming to the hospital. Values are no. abnormal/no. tested (%).  ¶p = 0.003, by χ^2^ test.

ELISA was performed on samples from 33 patients with acute Q fever and 18 patients in the control group. Blood samples were obtained from the study group at day 10 ± 8 of illness and from the control group at day 7 ± 6 of illness. Sensitivity of this ELISA was 61%.

CFA, which was performed for 81 patients in the study group at day 9 ± 19 of illness and for 52 patients in the control group at day 8 ± 6 of illness, showed a sensitivity of 22% (Table 5). A total of 57 patients were hospitalized, of whom 36 were given a diagnosis of acute Q fever during their hospitalization.

### Imaging Studies

A total of 78% of chest radiographs for patients with acute Q fever showed signs of pneumonia. A total of 54% of chest radiographs for patients in the control group showed signs of pneumonia (p = 0.003) (Table 5).

### Treatment

Treatment was started before a diagnosis was made. Significantly more patients with acute Q fever started treatment with doxycycline than patients in the control group (35% vs. 15%; p = 0.001) (Table 6). For 8 patients in the study group, the duration of antimicrobial drug treatment was unknown. Of the remaining 74 patients with acute Q fever, 34 (46%) patients were given adequate treatment. The mean ± SD follow-up time for patients given adequate treatment was 11.7 ± 5 months compared with 13.3 ± 9 months for patients given inadequate treatment.

**Table 6:** Initial treatment for patients with acute Q fever and control group with negative serologic results for Q fever, the Netherlands*

Initial treatment	Study group, n = 82, no. positive/no. tested (%)	Control group, n = 52, no. positive/no. tested (%)	p value†
Doxycycline	29/82 (35)	8/52 (15)	0.001
Moxifloxacin	5/82 (6)	2/52 (4)	NS
Ciprofloxacin	7/82 (9)	6/52 (12)	NS
Penicillin	7/82 (9)	1/52 (2)	0.049
Amoxicillin	13/82 (16)	5/52 (10)	NS
Amoxicillin/clavulanic acid	3/82 (4)	4/52 (8)	NS
Piperacillin/tazobactam	1/82 (1)	5/52 (10)	NS
Cephalosporin	14/82 (17)	17/52 (33)	NS
Co-trimoxazole	0/82 (0)	1/52 (2)	NS
Flucloxacillin	2/82 (2)	0/52 (0)	NS
Clarithromycin	0/82 (0)	1/52 (2)	NS
No treatment	1/82 (1)	1/52 (2)	NS
Unknown	0/82 (0)	1/52 (2)	NS
Patients with adequate treatment‡	34/74 (46)	NA	NA

*NS, not significant; NA, not applicable. †By χ^2^ test. ‡Defined as use of doxycycline (200 mg/d), moxifloxacin (400 mg 1×/d), or ciprofloxacin (500 mg 2×/d) for >2 wk.

### Outcomes

Hospitalization (70% vs. 94%; p = 0.001), admission to an intensive care unit (4% vs. 18%; p = 0.002), and need for respiratory support (2% vs. 16%; p = 0.001) were less common for the study group than for the control group (Table 7). Also, duration of hospital stay was shorter for patients with acute Q fever (9 ± 7 days vs. 17 ± 15 days; p = 0.001). Accurate follow-up data were available for 59 of 82 patients with acute Q fever who had a mean ± SD follow-up of 12.8 ± 8.2 months. Chronic Q fever developed in 6 (10%) patients in the Q fever group.

**Table 7:** Outcome, follow-up, and prophylaxis for patients with acute Q fever and control group with negative serologic results for Q fever, the Netherlands*

Characteristic	Study group	Control group	p value
Outcome			
Hospitalized	57/82 (70)	49/52 (94)	0.001†
Need for ICU	2/57 (4)	9/49 (18)	0.002†
Need for respiratory support	1/57 (2)	8/49 (16)	0.001†
Mean ± SD duration of hospitalization, d	9 ± 7	17 ± 15	0.001‡
Mean ± SD duration of time in ICU, d	5 ± 1	14 ± 10	0.266‡
Follow up			
Development of chronic Q fever	6/59 (10)	NA	NA
Development of long-lasting fatigue§	6/56 (11)	NA	NA
Death	5/82 (6)	10/52 (19)	0.019†
Q fever–related death	1/82 (1)¶	NA	NA
Indication for prophylaxis	16/82 (20)	NA	NA
Development of chronic Q fever			
Prophylactic treatment	0/8 (0)	NA	NA
No prophylactic treatment	3/6 (50)	NA	0.018#

*Values are no. positive/no. tested (%) unless otherwise indicated. ICU, intensive care unit; NA, not applicable. †By χ^2^ test. ‡By Student *t-*test. §Defined as persisting fatigue for >6 mo after acute Q fever in the absence of chronic Q fever. ¶This patient died of consequences of an infected vascular prosthesis caused by chronic Q fever. #By unilateral Barnard exact test.

Sixteen patients with acute Q fever met the criteria for prophylactic treatment to prevent development of chronic Q fever (Table 8). Indications were valvular dysfunction (n = 8); cardiac valve prosthesis (n = 3); aneurysm (n = 1); vascular prosthesis (n = 3, of whom 1 patient also had a cardiac valve prosthesis); and a new cardiac murmur (n = 2). Eight (50%) of these patients received prophylactic treatment. Proper follow-up data for development of chronic Q fever were available for 14 patients with an indication for prophylaxis. Chronic Q fever did not develop in any of the 8 patients who received prophylaxis. The other 6 patients with an indication for prophylaxis for whom follow-up serum samples were available did not receive prophylaxis because the indication for prophylaxis was not recognized by the treating physician. Chronic Q fever developed in 3 (50%) of these 6 patients (p = 0.02). In the group without an indication for prophylaxis, chronic Q fever developed in 3 (6%) patients. Six (11%) of 56 patients in the study group for whom these data were available reported long-lasting fatigue.

**Table 8:** Characteristics of 16 patients with acute Q fever with an indication for prophylaxis, the Netherlands*

Patient no.	Age, y/sex	Hospitalized	Indication at presentation for prophylactic treatment	Prophylactic treatment and duration, mo	Chronic Q fever	Died
1	42/M	Yes	Valvular dysfunction (AS)	D + H, 12	No	No
2	49/M	Yes	Cardiac bioprosthesis and vascular prosthesis	D + H, 12	No	No
3	51/M	Yes	Cardiac bioprosthesis and TOF	D 12 + H 4 (added after 8)	No	No
4	54/M	Yes	Aneurysm common iliac artery	D + H, 9	No	No
5	43/M	Yes	Valvular dysfunction (TI) and TGV	D + H, 7	No	No
6	78/F	Yes	Cardiac bioprosthesis	D + H, 1, switched to Mox, 3	No	Yes†
7	26/M	No	Vascular prosthesis	D + H, 2.5	No	No
8	81/F	Yes	Valvular dysfunction (MI)	D + H, 12	No	Yes‡
9	65/M	Yes	Valvular dysfunction (MI)	No	No	No
10	80/M	Yes	Valvular dysfunction (MI)	No	No	No
11	78/F	No	Valvular dysfunction (MI)	No	No	No
12	64/F	Yes	Vascular prosthesis	No	Yes	Yes§
13	75/F	Yes	New cardiac murmur	No	Yes	No
14	75/M	No	New cardiac murmur	No	Yes	No
15	57/F	No	Valvular dysfunction (AS)	No	Unknown¶	No
16	58/M	Yes	Valvular dysfunction (MI)	No	Unknown¶	No

*AS, aortic valve sclerosis; D, doxycycline 100 mg 2×/d; H, hydroxychloroquine 200 mg 3×/d; TOF, tetralogy of Fallot; TI, tricuspid insufficiency; TGV, transposition of the great vessels; Mox, moxifloxacine 400 mg 1×/d; MI, mitral insufficiency; CFA, complement fixation assay; IFA, immunofluorescence assay. †This patient was rehospitalized shortly after the acute Q fever episode and died because of a reason unrelated to Q fever. The last available serologic follow-up showed no signs of chronic Q fever (negative PCR result; CFA titer 1:10, IFA IgG phase I negative result; IgG phase II titer 1:256; IgM phase I negative result; and IgM phase II titer 1:64). ‡This patient eventually died because of a reason unrelated to Q fever. The last available serologic follow-up 1 year after the acute Q fever episode showed no signs of chronic Q fever (negative PCR result; CFA titer 1:10; IFA IgG phase I titer 1:64; IgG phase II titer 1:512; IgM phase I titer 1:16, and IgM phase II titer 1:16). §This patient was hospitalized and admitted to the intensive care unit for 5 d. She was treated with several antimicrobial drugs (penicillin, ciprofloxacin, cefuroxim, metronidazol, ceftazidim, and teicoplanin) before given a diagnosis of an infected vascular prosthesis caused by chronic Q fever. Although doxycycline and hydroxychloroquine were given after the diagnosis was made, this patient eventually died from consequences of an infected vascular prosthesis caused by chronic Q fever. ¶No follow-up with reference to Q fever was performed for this patient.

The mortality rate during a 12-month follow-up period was 6% for the study group compared with 19% for the control group (p = 0.02). None of the patients in the study group died during the episode of acute Q fever. Four patients in the study group died because of reasons unrelated to Q fever. One patient died of consequences of an infected vascular prosthesis caused by chronic Q fever, although adequate treatment was started after the diagnosis. In contrast, 2 patients in the control group died during initial hospitalization, 1 of a *Mycoplasma* sp. infection and 1 of pneumonia without a known causative agent. Eight patients in the control group died during follow-up. One of them died of a non-Hodgkin lymphoma and 1 of consequences of an *Aspergillus* sp. infection. For the other 6 patients who died, no detailed information was available.

A total of 49 control patients were given a diagnosis of pneumonia; for 38 of these patients, no causative agent was found. For the remaining 11 patients, causative agents were *Pneumocystis jiroveci*, *Moraxella catarrhalis*, *Legionnella pneumophila*, *Chlamydia* sp., *Haemophilus influenzae* (2 patients), *Mycoplasma* sp. (3 patients), influenza virus and *Mycoplasma* sp., and *Staphylococcus aureus* and *Streptococcus pneumonia*. The remaining 3 patients were given diagnoses of acute myeloid leukemia, non-Hodgkin lymphoma, and restrictive pericarditis.

## Discussion

This retrospective case–control study evaluated differences in clinical signs and symptoms between patients with acute Q fever referred to a hospital and a control group. Because patients in the control group had Q fever included in the differential diagnosis, a selection bias is possible. However, differences were found between the 2 groups. In addition, because of the Q fever outbreak during that time, *C. burnetii* was considered a possible etiologic agent in many patients who came to a hospital. The higher number of patients in the study group can be explained by strict implementation of inclusion criteria for the control group.

Consistent with findings of earlier studies (*1**,**21*), we found that patients with acute Q fever more often had a history of smoking. However, a history of lung disease was found less often. A lower mean age in the study group than in the control group might explain this finding. Previous studies suggest typical signs and symptoms of acute Q fever: fever, headache, and cough (*1**,**3**,**22*). However, no difference was observed in the occurrence of fever. It has been postulated that headache is rather specific for acute Q fever (*5**,**23*). However, in our study, headache was less common in patients with acute Q fever than in the control group. Although cough was a relatively common sign in both groups, sputum production was reported less often in patients with acute Q fever. In addition, a sore throat was reported more often in the study group, which has not been previously reported.

A limitation of these results is the retrospective nature of the study because physicians probably did not include all signs and symptoms in patient charts. In general, patients with lung disease often use corticosteroids, which might explain why fewer patients in the study group were classified as immunocompromised. In contrast to medical and physical examination results, more patients with acute Q fever showed signs of an infiltrate on chest radiographs when they came to the hospital. Although acute Q fever usually is a relatively mild influenza-like disease, it has been reported that chest radiographs often shows signs of an infiltrate (*24*). Compared with our control group, fewer patients in the study group needed hospitalization, and duration of hospitalization was shorter. These findings might be explained by the lower mean age of patients with acute Q fever, assuming that they were in a more healthy condition. Furthermore, *C*. *burnetii* is known for its self-limiting character, in contrast to those of other pathogens found in the control group.

In the Netherlands, a Q fever hospitalization rate of 50% in 2007 was registered, which stabilized at ≈20% in later years (*25*). This rate is higher than that previously reported (2%–5%) (*5*). However, large variations in hospitalization rates for acute Q fever patients have been reported (*26*). In this study, 70% of patients with acute Q fever were hospitalized. Most patients with acute Q fever are asymptomatic or have only a mild influenza-like illness. Thus, a selection bias caused by the study design is likely. We found that 78% of patients in the study group had an abnormal result on a chest radiograph, which might indicate that only patients with severe symptoms were hospitalized.

Although C-reactive protein levels and leukocyte counts differed between the study group and the control group, this finding did not contribute to differentiation between *C. burnetii* and other pathogens at hospitalization because differences were small and showed much overlap. In addition, although leukocyte counts were usually within the reference range, patients with acute Q fever more often had a lower leukocyte count, which is consistent with results of other studies (*3**,**4*). In contrast to these studies, which found thrombocytopenia in patients with acute Q fever, we found slightly higher levels of platelets, all within the reference range, in the study group than in the control group. Increased levels of liver enzymes have been reported in patients with acute Q fever (*3**,**5**,**22*). However, we found no differences in these levels between both groups at hospitalization. Furthermore, creatinine levels were not increased, in contrast to results reported in a previous study (*3*).

Although antimicrobial drug treatment was inadequate in an unexplainably high percentage of patients with acute Q fever, more patients in the study group than in the control group were initially treated with doxycycline, the treatment of choice for patients with acute Q fever. The choice of antimicrobial drug treatment in patients with community-acquired pneumonia (CAP) of unknown origin in the Netherlands depends on the Confusion, Urea nitrogen level in blood, Respiratory rate, Blood pressure, age >65 years (CURB-65) score (*27*). In addition, although CURB-65 scores could not be calculated for all patients, fewer patients in the study group were hospitalized, needed admission to an intensive care unit, and needed respiratory support, which suggests lower CURB-65 scores in the study group than in the control group.

Although changes were made in the national guidelines for treating CAP issued by the Dutch Working Party on Antibiotic Policy in 2011 (*28*), until 2011, doxycycline was the first choice for patients with a low CURB-65 score (*29*). In addition, more patients in the study group were given a diagnosis of having an infiltrate, which suggested that initial treatment in the study group was also aimed at atypical microorganisms. Presumably, patients in the control group were treated with broader spectrum antimicrobial drugs because of higher CURB-65 scores. Also, more patients in the control group were immunocompromised, which also could have influenced the choice of treatment.

Long-term prophylactic treatment with doxycycline and hydroxychloroquine has been suggested for patients with risk factors for development of chronic Q fever (*12**,**14*). Although controversy still exists (e.g., with regard to treatment duration and patient selection), prophylactic treatment of high-risk patients after an episode of acute Q fever can be beneficial and is widely advised (*30**–**32*). In our study, not all patients who had an indication according to our definition received prophylaxis. Chronic Q fever developed in 3 of 6 patients who did not receive prophylaxis, in contrast to none of the patients who received prophylaxis, which is a difference that clearly supports findings of other studies in which prophylactic treatment was suggested to prevent development of chronic Q fever in patients with risk factors for this disease (*12**,**14*). On the basis of these results, prophylactic treatment is advised if risk factors for developing chronic Q fever exist, but potential side effects must be taken into consideration (*33*).

For 48 of 67 patients without indication for prophylactic treatment, follow-up data were available on development of chronic Q fever. Chronic Q fever developed in 3 (6%) of these patients, which is slightly higher than expected (*1**,**34*). This finding might be explained by the fact that we included only patients who were referred to a hospital, and therefore selected patients most affected by *C*. *burnetii* infection. It is possible that more severely acute Q fever predisposes for development of chronic Q fever (*13*).

After having acute Q fever, patients often report long-lasting fatigue, which frequently persists for >6 months. This symptom after acute Q fever has been designated Q fever fatigue syndrome. Our data suggest a prevalence of 11%, which is lower than expected; other studies reported a prevalence of ≈20% worldwide and a higher prevalence in the Netherlands (*6**,**35**,**36*). The prevalence found in this study is presumably an underestimation because proper analysis was not performed for most patients.

Although we found some differences in clinical manifestations for patients with acute Q fever coming to a hospital compared with controls, considerable overlap between both groups hamper the use of these variables for clinical differentiation. Differentiating *C*. *burnetii* from other pathogens is not possible without Q fever serologic analysis and PCR in patients coming to a hospital. In disease-endemic areas or in instances in which patients have risk factors for Q fever, suspicion should remain high, and the threshold for performing Q fever serologic analysis and PCR should remain low. Because only 46% of patients received adequate treatment acute Q fever in our study, treatment for acute Q fever should be improved. Furthermore, our findings underline the recommendation that prophylactic treatment should be given to patients with risk factors for developing chronic Q fever. However, more studies are needed to develop uniform guidelines with regard to optimal prophylactic treatment.
